# Patterns of attendance to health checks in a municipality setting: the Danish ‘Check Your Health Preventive Program’^☆^

**DOI:** 10.1016/j.pmedr.2016.12.011

**Published:** 2016-12-21

**Authors:** Anne-Louise Bjerregaard, Helle T Maindal, Niels Henrik Bruun, Annelli Sandbæk

**Affiliations:** a Section of General Practice, Department of Public Health, Aarhus University, Aarhus, Denmark; b Steno Health Promotion Centre, Steno Diabetes Center A/S, Gentofte, Denmark; c Section of Health Promotion and Health Services, Department of Public Health, Aarhus University, Aarhus, Denmark; d Research Unit of General Practice, Aarhus University, Aarhus, Denmark

**Keywords:** Health checks, Health examinations, Attendance, Prevention

## Abstract

We aimed to investigate the determinants of attendance to a preventive health check program and to explore the homogeneity of the attenders.

4853 eligible persons living in the municipality of Randers, Denmark, from 2012 to 2013, aged 30–49 years, received an invitation to attend the ‘Check Your Health Preventive Program’. Data was obtained from the Danish National Registers. Socio-demographic factors, use of preventive services, morbidity were examined as determinants of attendance by Poisson regression analyses. A chi-squared automatic interaction detection decision tree analysis was used to identify mutually exclusive groups.

In total, 55% of the invited population attended (49% men). Attenders were more likely to be: of higher age; immigrants; cohabiting; have: higher socio-economic status; higher use of preventive services and lower morbidity. Decision tree analysis revealed six groups, with the most important variable being income: 1) low income, low education (A = attendance rate: 38%; P = population size: 11%); 2) low income, education higher than 10 years, living alone (A: 41%; P: 5%); 3) low income, education higher than 10 years, cohabiting (A: 56%; P: 16%); 4) middle income (A: 60%; P: 34%); 5) high income, living alone (A: 56%; P: 4%); 6) high income, cohabiting (A: 69%; P: 30%).

More than half of a general population voluntarily attended a general health check, despite a resource intensive offer. People with low resources had lower attendance rates. This study adds a detailed description of mutually exclusive groups of attenders, for use in future planning and implementation of preventive actions.

## Highlights

• Attendance was determined by socio-demographic factors and preventive services use.
• Morbidity and medication purchase was associated with lower attendance rate.
• Decision tree analysis revealed six groups of attenders.
• Groups with low income, educational level, living alone, had lower attendance rate.
• Targeted recruitment strategies should be considered as a way to increase attendance.

## 1 Introduction

Early detection of persons at risk is crucial in order to reduce the risk for development of chronic disease. One way of identifying persons at risk is to invite the general population to attend intervention- (Jørgensen et al., 2003, Kaczorowski et al., 2008) and screening programs or to engage in regular health visits at their general practitioner (Krogsbøll et al., 2012, Simmons et al., 2012). Regular health examinations are systematically offered in the UK, in the NHS Health Check program (Cochrane et al., 2012) and in the US (Han, 1997). However, it is inconclusive if regular health examinations conducted by general medical practices reduces mortality and morbidity rates (Krogsbøll et al., 2012). A well-known challenge of screening and systematic health checks is low uptake (Krogsbøll et al., 2012) which seems to be influenced by a number of factors (Dryden et al., 2012, Bender et al., 2012). A higher participation rate has, in many studies, been found to be associated with higher age (Jones et al., 1993, Waller et al., 2013, Artac et al., 2013), being a woman (Waller et al., 2013, Culica et al., 2002, Thomas et al., 1993), and with socio-demographic factors such as having a job; higher educational level; higher income; and living with a partner (Bender et al., 2012, Boshuizen et al., 2006, Hoebel et al., 2014). Only a few studies have investigated the association of people's morbidity with non-attendance in general health examinations (Dryden et al., 2012, Boshuizen et al., 2006, Thorogood et al., 1993), since it is difficult to obtain records of disease for those who do not want to participate. Morbidity seems to influence participation in health examinations and screening studies but the associations point in different directions (Wall and Teeland, 2004, Karwalajtys et al., 2005).

Whether or not the same patterns of attendance are seen in a general Danish population (aged 30–49 years) that was invited to preventive health examinations by their general practitioner is unclear. It is important to know whether there are factors and personal characteristics that increase likelihood of attending health checks. Additionally, it is important in order to be able to adjust and tailor future invitation procedures to the right recipients (e.g. high risk population).

By using data from the ‘Check Your Health Preventive Program’ (CHPP) and from Danish national registers, we aimed to investigate the determinants of attendance to a preventive health check program and to explore the homogeneity of the attenders.

## 2 Methods

### 2.1 Study design and population

A cohort study was performed based on exposure data from the Danish National registries (2011) and data on attendance from the first year (April 2012–May 2013) of the Danish ‘Check Your Health Preventive Program’ (CHPP) (Maindal et al., 2014). The CHPP is a prevention and health promotion program running in a municipality-led Health Care Centre in Randers, Denmark. The core components of the program is health examinations followed by supportive consultations performed in a close collaboration between general practice and the municipality setting (Maindal et al., 2014). Citizens, aged 30–49 years, living in the municipality of Randers on January 1, 2012 (N = 26.216), were randomized into five groups and invited to attend the CHPP during the five consecutive years 2013–2017. The randomization was a cluster randomization based on households identified by addresses obtained from the Danish Civil Register (Maindal et al., 2014). During the first year of the CHPP, 5261 citizens were randomly selected for invitation to participate. Of these, 20 participants withdrew their consent and hence were excluded from the study. Another 226 persons who had died or migrated from the municipality of Randers in between the randomization date and invitation date was excluded. Furthermore, 116 persons were excluded, since their general practitioner refused participation in the study. Thus, the total population (*N*) comprised 4853 citizens who were randomized and received an invitation to participate in the health check program in the period from 18th of April 2012 to 30th of May 2013. Invitations were sent out by mail and included an invitation letter, information leaflet, a questionnaire, and a pre-booked date and time for the health examination. People living together were offered an examination on the same date. Pre-booked appointments could be postponed in case of pregnancy, planned operations, illness, having had a recent health examination by the general practitioner or other similar reasons. People who did not respond to the invitation were sent a reminder.

### 2.2 Exposures

Data from the Danish national registries (Statistics Denmark, www.dst.dk) were obtained for the population (n = 4853), for the year 2011. The unique Danish civil registration number was used to merge information from the registers to information from the health checks.

### 2.3 Socio-demographic variables

Socio-demographic variables included education, occupation, income, cohabitation status, and ethnicity. Education was defined as the highest formal educational attainment and was classified according to the UNESCO categories (UNESCO Institute for Statictics, 2011) and categorized into three groups: < 10, 10–15, and > 15 years of education. Occupation was categorized into four groups: being employed, self-employed, unemployed, or social welfare recipients. OECD-adjusted income level (OECD Project on Income Distribution and Poverty, 2011) was defined using the family's disposable income, adjusted for family size and categorized into tertiles (low/middle/high income). Cohabitation status was defined as cohabiting or living alone (i.e. one person in household). Ethnicity was categorized into three groups: immigrants (persons not born in Denmark, parents were not Danish citizens and not born in Denmark), descendants (persons born in Demark, parents were not Danish citizens and not born in Denmark ) or Danish (rest of the population).

### 2.4 Morbidity

Disease status was obtained based on ICD10 diagnosis codes (ICD-10 codes: hypertension [DI11 to DI15], Diabetes Mellitus [DE10 to DE14], hypercholesterolaemia [DE78], asthma [DJ45 and DJ46], Mental disease [DF]), provided by the ‘National Patient Registry’, and on medicine usage, obtained from the ‘Register of Medicinal Product Statistics’. A binary morbidity score was created (yes/no), indicating the presence (or absence) of one or more of the above listed diseases or medication purchased.

### 2.5 Use of preventive health services

In Denmark, every citizen is attached to a general practitioner (GP), and 85% of all citizens see their GP once a year (Moth et al., 2012). The National Health Service covers any costs related to GP consultation or hospital-admissions, and furthermore part of the costs of one yearly visit to the dentist. Information on number of visits to the dentist during the last five years was obtained from the ‘National Health Insurance Service Registry’ and classified as: zero to one visits, two to four visits, and five or above visits.

### 2.6 Outcome

Health examination attendance was categorized into three exclusive groups: (i) attending the health examination (attenders), (ii) actively declined participation (active non-attenders), or (iii) not attending/not declining the health examination (non-attenders).

### 2.7 Statistical analysis

Attendance rate was defined as the percentage of citizens attending the health examination of the total population (total population: total number of citizens randomized and invited to the first year of the CHPP). Characteristics of attenders and non-attenders are presented (Table 1) and compared using the chi-square test for comparison of proportions. Level of significance was set to 5%.

Poisson regression models with robust error variance were used for estimating relative risks associated with attendance (Table 2, Table 3). For that purpose, the ‘active non-attenders’ were excluded from the analysis. Analyses were adjusted for confounders according to the literature (Bender et al., 2012, Jensen et al., 2013). The model on age was adjusted for sex, educational attainment, occupational status and income. Ethnicity and cohabitation were adjusted for age, sex, educational attainment, occupational status and income. Educational attainment was adjusted for age and sex only, since it is not confounded by other socioeconomic factors. The model on income was adjusted for age, sex, educational attainment, occupational status and cohabitation status. Occupational status was adjusted for age, sex, educational attainment and morbidity. Models including morbidity and use of preventive health services were adjusted for age, sex, educational attainment, occupational status and income. The relative risk (RR) of attendance (with 95% confidence intervals, CI) from age and sex-adjusted models are presented as well as results from fully adjusted models (Table 2, Table 3).

A chi-squared automatic interaction detection (CHAID) decision tree analysis was performed to explore data homogeneity, to identify relationships between variables, and, to verify whether they are associated to attendance (Kass, 1980). In decision tree analysis, information is grouped into mutually exclusive subsets based on homogeneity of the data. In brief, a series of chi-square tests are performed to achieve optimal splits into nodes within each categorical determinant with respect to the attendance variable. Likewise (based on chi-squares) the most optimal determinant is chosen. The algorithm is continued as long as nodes/splits do not become too small. All potential determinants of attendance were used as categorical input for the CHAID analysis: age, sex, ethnicity, cohabitation status, occupational status, education, income, and morbidity. The CHAID analysis was run with parent nodes defined at a minimum of 200 persons, child nodes defined at a minimum of 100 persons, and significance (α_merge_, α_split_, and *P*-value) set at ≤ 0.05. All analyses were performed using STATA statistical software version 14 (StataCorp, 2015).

### 2.8 Ethical considerations

The present study did not need ethical approval, since it includes existing data obtained for the CHPP only. The CHPP complied with the Helsinki Declaration and written informed consent to use data for research purposes was obtained for all participants prior to their health examination. The Danish Data Protection Agency approved the storing of data (ref. no. 2013-41-2511).

## 3 Results

**Fig. 1:**
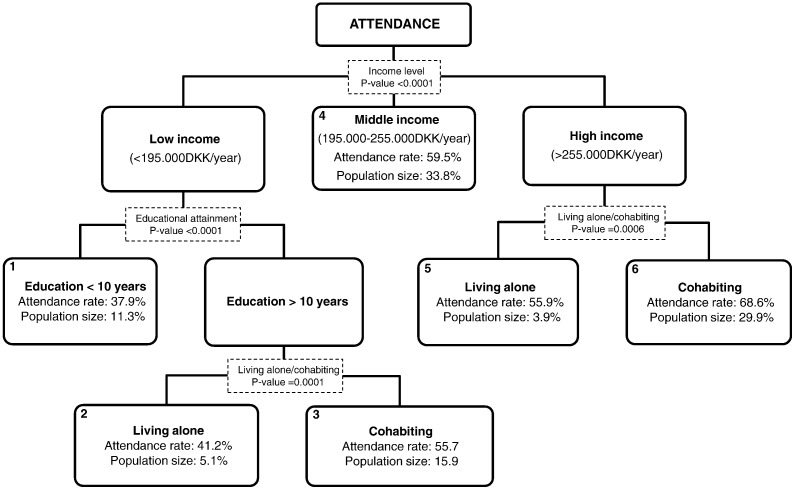
Classification tree of determinants of attendance to the Check Your Health Preventive Program. The decision tree analysis depicted six distinct groups of attenders. Attendance rate indicates the percentage of attenders in the respective group of 4538 invited persons with complete data in all variables included in the model. Population size indicates the group population proportion of the 4538 persons with complete data. Dotted boxes indicate the statistical significant variables causing the split.

In total, 55% of the invited 4853 persons attended a health check. Median age of attenders was 41.5 (1st Qrt: 36.5; 3rd Qrt: 45.8) years. 49% were men. The median time from invitation to examination date was 38 days (range: three to 1181 days). 95% attended within 102 days after invitations were send out and 1% attended between 499 and 1181 days. Five percent actively declined the offer (Table 1). In total, 40% of the invited did not attend. A description of attenders, active non-attenders and non-attenders are given in Table 1, while relative risk differences for attendance are given in Table 2, Table 3. Decision tree analysis showed that attenders were not a homogenous group of people. Six groups of attenders were found categorized uniquely by values of income, cohabitation status and education level (Fig. 1).

### 3.1 Age, gender and ethnicity

Among the 2482 invited men, 53% attended the health check, while 57% of the 2371 invited women attended (Table 1). Higher attendance was seen in persons of higher age, and among immigrants (Table 2).

### 3.2 Sociodemographic characteristics

Persons living alone were more likely not to attend the health check, as compared to persons cohabiting. Likewise, a significant higher attendance rate was seen in persons having a partner included in the study (Table 2). As compared to being employed, being unemployed or receiving social welfare was associated with lower RR of attendance. Furthermore, having the lowest income level and lowest level of educational attainment was associated with lower attendance (Table 2).

### 3.3 Disease burden

Data from the national registries showed that 1235 (25%) of the invited had one or more of the following diseases: hypertension, diabetes, hypercholesterolemia, asthma or mental disease symptoms or purchased medicine for the same conditions. Morbidity was associated with a RR of 0.88 (0.83; 0.94) of attendance to the health check (Table 2). As compared to not having purchased medicine during the last year, having purchased medicine (except from antiasthmatic medicine) was associated with a 8–40% risk reduction in attendance, when adjusting for gender and age. After additionally adjustments for occupational status, education and income, the RR remained significantly lower for those having purchased medication for diabetes, antihypertensives, and antipsychotics (Table 3).

### 3.4 Preventive services

Nearly 80% of the invited had five or more visits to the dentist during the last five years before invitation and 13% had no contact to dentist. Having five or more visits to dentist in the five years before invitation was associated with a significantly higher attendance rate to health checks with a RR of 1.53 (1.37; 1.71), compared to persons who had no visits (Table 2).

### 3.5 Classification of attenders

As shown in Fig. 1, the decision tree analysis revealed six distinct groups: 1) people with low income level and education < 10 years (A = attendance rate: 38%; P = population size: 11% of the total attending population with complete data, n = 4538); 2) low income level, > 10 years of education and living alone (A: 41%; P: 5%); 3) low income level, > 10 years of education and cohabiting (A: 56%; P: 16%); 4) middle income level (A: 60%; P: 34%); 5) high income level, living alone (A: 56%; P: 4%); and 6) high income level, cohabiting (A: 69%; P: 30%).

The main determinant of attendance to health check was income. For those with the highest income level, cohabiting was the second most important determinant of attendance. For those with the lowest income level, longer educational attainment (> 10 years) was the second strongest determinant. Among those, who had the lowest income level albeit > 10 years of education, cohabitation was the third strongest determinant of attendance (Fig. 1).

## 4 Discussion

This study showed that 55% of those invited attended the ‘Check Your Health Preventive Program’ in the first year running. In total, 40% did not respond to the invitation, whereas 5% actively declined participation. The attendance rate is in line with those reported in other European studies. Furthermore, our findings confirm those of previous studies; that the relative risk of attending the health check was higher in persons of higher age, with higher educational attainment, and more use of preventive services. In contrast, factors associated with a reduction in the relative risk of attending were: living alone, receiving social welfare or being un-employed, and morbidity (Artac et al., 2013, Hoebel et al., 2014, Fossa et al., 2015, Tolonen et al., 2015, Hoebel et al., 2013). The findings on morbidity and treatment are novel and provide a detailed picture of the diagnosis and treatment status and how this is associated with attendance. Decision tree analysis suggested six mutually exclusive groups of attenders, with income being the strongest determinant of attendance. The majority of the attenders had middle and high income level and was cohabiting (group 4 and 6, Fig. 1). The largest potential for increasing attendance was seen in groups of people with low income and low education and in groups of people who were living alone (group 1, 2 and 5, Fig. 1). Low social status, as measured by low educational level, low income and unemployment has earlier been associated with a lower participation rate (Bender et al., 2012, Artac et al., 2013, Hoebel et al., 2013, Jensen et al., 2012, Cochrane et al., 2013, Dalsgaard et al., 2009). The underlying mechanisms are not fully understood, but educational attainment might influence a person's health literacy and thus the ability to understand the invitation material and the importance of early disease detection (Bo et al., 2014). For income, it has been suggested, that persons with a low income tends to worry more about their financial problems rather than their future health problems (Hoebel et al., 2013). As a consequence, they might not participate in preventive health checks. The variable ‘living alone’ can be viewed as a proxy for low social support. People living alone do not have the same amounts of contacts to the rest of the society (as for instance people living together with friends or family). Poor social support has been found to be associated with a lower participation rate (Hoebel et al., 2014) and loneliness and living alone is (together with poor social network) associated with poorer health status (Rico-Uribe et al., 2016). Together with our positive finding of inviting spouses together, this suggests that other ways of recruiting people, for instance through social networks (family, friends, clubs, societies, or at work) might work as a way of increasing participation rate in health checks.

We found an interplay between the sociodemographic determinants in their prediction of attendance, so that one determinant is not independent of another. The finding means that future participants can be classified based on a short list of characteristics to minimize administrative burden (Linden and Yarnold, 2016), and thus, they are rather simple to identify. For instance, the group with low income and low educational attainment comprised 514 persons. Of these, 62% did not attend the health check during one year. Knowing that this group constitutes one of the most burdensome populations concerning chronic diseases and inappropriate health behavior, a brief intervention focused at this group could be added to the general invitation, e.g. a personal contact by telephone or a visit.

The finding that morbidity and treatment with medication for diabetes, antihypertensives, and antipsychotics was associated with lower attendance can be interpreted as a natural self-selection for health checks. People who visit their general practitioner because of illness might refuse health examinations because they are already being checked (Wall and Teeland, 2004), or might not participate due to low surplus energy. This hypothesis is supported by findings from NHS (Jenkinson et al., 2015). People with a higher use of preventive services (e.g. visits to dentists, participation in screening programs, compliance to child vaccinations or having routine examinations at the general practitioner) itself, has been found to be more likely to participate in health examinations (Boshuizen et al., 2006, Wall and Teeland, 2004, Cherrington et al., 2007). This is in line with our finding that those with regular visits to the dentist were more likely to attend the health check, indicating a higher degree of responsibility towards their own health. Our finding that a higher proportion of immigrants than ethnic Danes attended the study is in line with the finding from NHS (Artac et al., 2013) but different from another large Danish study (Ahlmark et al., 2014). However, the latter study examined non-western immigrants in comparison to ethnic Danes, whereas we examined immigrants as a total. Previously, immigrants have been found to have a higher contact pattern to the general practitioner as compared to ethnic Danes, and the higher proportion of immigrants attending the health examination might thus be due to culturally differences in health-seeking behavior (Norredam, 2011).

### 4.1 Strengths and limitations

A major strength of our study is the representative study population. The persons invited to the first year of the CHPP were citizens, aged 30–49 year old, living in Randers Municipality in 2012–2013. It was not just a subset of the population, or persons covered by health insurance, as seen in other studies. Randers is the sixth largest city in Denmark and is part of a larger region (‘Central Denmark Region’) comprising 19 municipalities. As compared to the national level, the citizens in Central Denmark Region have roughly the same estimates of self-rated health, social support, social network, health behavior and disease burden (Sundhedsstyrelsen, 2013). The citizens in Randers differs from those in Central Denmark Region by having more passive transportation to and from job, an unhealthy diet, and higher proportions of persons with heavy overweight and stress-related diseases (Larsen et al., 2014). The results can be generalized to the general population in Denmark (aged 30–49 years), although one may speculate higher participation rates in a national context. The program allowed citizens to postpone their pre-booked appointment (see Methods Section). Since the program is a five-year program running in the municipality, we included no restrictions on how long an appointment could be postponed. Attendance rates are expressed according to time. The majority of the attenders in CHPP underwent the health examination within one year. Only 26 citizens participated from 1.4 to 3.2 years after invitation, and thus, our results are comparable to other studies reporting attendance rates over one-to-two years of examination (Bender et al., 2012, Artac et al., 2013). The use of the national registries to obtain data as well as the actual rate of participation in the CHPP study, rather than self-report measures, minimized selection and information bias and enhanced the validity of the study. This furthermore allowed us to include a range of explanatory variables, with only few missings. Potential confounding factors identified in the literature were included in the analysis, but residual confounding cannot be eliminated.

Another strength of this study is the use of decision tree analysis to classify the participants into mutually exclusive groups. The analysis allowing for the examination of interactions is based on a data driven perspective rather than a priori assumptions hypothesized to be important based on existing research. Thus, the decision tree methodology provides an insight into the underlying relationship of the independent variables in predicting the outcome, giving a more detailed picture. Using classification tree methods has been found to be more accurate than other classification methods in classifying participants and non-participants (Linden and Yarnold, 2016). The main limitation of decision tree analysis is that the variables in the model may not be the only important ones. However, other statistical methods (such as regression analysis) are affected by multicollinearity as well.

## 5 Conclusion

People with low resources, higher disease burden, and unbeneficial health behaviors such as not having regular visits at their dentist, have lower attendance rates to general health checks. Remarkable is also that more than half of a general population voluntarily turn up, despite a resource intensive offer. This study adds a detailed description of mutually exclusive groups of attenders. The lowest attendance rates were seen in people with low income level and low education level, and in groups of people living alone. Individual approaches to invitation procedure and to prevention may be considered for groups with low attendance, without high costs, by targeting needs, resources, and health literacy in the planning and implementation of health checks.

## Conflicts of interest

None declared.

## Transparency document

Transparency document.

## Figures and Tables

**Table 1:** Characteristics of attenders and non-attenders invited to the Check Your Health Preventive Program 2012–2013.

	Attenders	Non-attenders	Active non-attenders	Total	*P*-value^a^	Missings
Numbers, n (pct)	2679 (55.2)	1949 (40.2)	225 (4.6)	4853		0/4853

Socio-demographic variables						
Gender, n (row pct)						0/4853
Woman	1354 (57.1)	887 (37.4)	130 (5.5)	2371		
Man	1325 (53.4)	1062 (42.8)	95 (3.8)	2482	0.0001	
Age, n (row pct)						0/4853
30-	488 (49.5)	458 (46.5)	39 (4.0)	985		
35-	644 (52.1)	523 (42.3)	70 (5.7)	1237		
40-	730 (56.3)	514 (39.6)	53 (4.1)	1297		
45-	817 (61.2)	454 (34.0)	63 (4.7)	1334	0.0000	
Ethnicity, n (row pct)						0/4853
Danish	2479 (55.1)	1815 (40.3)	207 (4.6)	4501		
Immigrants	200 (56.8)	134 (38.1)	18 (5.1)	352	0.6772	
Living alone, n (row pct)						0/4853
No	2298 (57.8)	1500 (37.7)	180 (4.5)	3978		
Yes	381 (43.5)	449 (51.3)	45 (5.1)	875	0.0000	
Partner in project (Yes), n (row pct)	1754 (58.0)	1129 (37.3)	142 (4.7)	3025	0.0000	0/4853
Occupational status, n (row pct)						1/4853
Employed	2219 (58.4)	1426 (37.5)	156 (4.1)	3801		
self-employed	127 (60.5)	78 (37.1)	5 (2.4)	210		
Unemployed/benefits	98 (43.0)	115 (50.4)	15 (6.6)	228		
Social welfare recipients	194 (37.9)	277 (54.1)	41 (8.0)	512		
Others	40 (39.6)	53 (52.5)	8 (7.9)	101	0.0000	
Education (years), n (row pct)						96/4853
< = 10	407 (43.5)	479 (51.2)	49 (5.2)	935		
10–15	2069 (58.0)	1346 (37.7)	154 (4.3)	3569		
> 15	161 (63.6)	78 (30.8)	14 (5.5)	253	0.0000	
Income, n (row pct)						6/4853
Low tertile	720 (44.8)	806 (50.1)	82 (5.1)	1608		
Middle tertile	924 (57.2)	628 (38.9)	64 (4.0)	1616		
High tertile	1035 (63.8)	510 (31.4)	78 (4.8)	1623	0.0000	

Disease burden						
Morbidity (Yes), n (row pct)	585 (47.4)	565 (45.7)	85 (6.9)	1235	0.0000	0/4853

Use of preventive services						
Number of visits to dentist the last 5 years, n (row pct)						27/4853
0–1	230 (35.8)	378 (58.9)	34 (5.3)	642		
2–4	112 (35.7)	188 (59.9)	14 (4.5)	314		
≥ 5	2329 (60.2)	1368 (35.3)	173 (4.5)	3870	0.0000	

a *P*-value from Chi-square tests.

**Table 2:** Relative risks and 95% confidence intervals (CI) for attendance to the Check Your Health Preventive Program (2012 − 2013), estimated by Poisson regression with robust error variances.

	Model 1			Model 2		
Determinant	N	Attenders (%)	Relative risk (95% CI)	N	Attenders (%)	Relative risk (95% CI)
Age			adjusted For sex			Adjusted for sex, education (years), occupational status, income
30-	946	51.59	1.00 (–; –)	908	51.98	1.00 (–; –)
35-	1167	55.18	1.07 (0.99; 1.16)	1146	55.15	1.05 (0.97; 1.14)
40-	1244	58.68	1.13 (1.05; 1.23)	1228	58.79	1.13 (1.04; 1.22)
45-	1271	64.28	1.25 (1.16; 1.34)	1256	64.57	1.21 (1.12; 1.30)
Ethnicity			Adjusted for sex, age			Adjusted for sex, age, education (years), occupational status, income
Danish	4294	57.73	1.00 (–; –)	4275	57.85	1.00 (–; –)
Immigrants	334	59.88	1.05 (0.96; 1.15)	263	62.36	1.24 (1.13; 1.37)
Living alone			Adjusted for sex, age			Adjusted for sex, age, education (years), occupational status, income
No	3798	60.51	1.00 (–; –)	3731	60.71	1.00 (–; –)
Yes	830	45.90	0.77 (0.71; 0.84)	807	46.10	0.86 (0.79; 0.93)
Partner in project			Adjusted for age			Adjusted for age, education (years), occupational status, income
No	1745	53.01	1.00 (–; –)	1695	53.22	1.00 (–; –)
Yes	2883	60.84	1.16 (1.10; 1.22)	2843	61.03	1.06 (1.00; 1.12)
Occupational status			Adjusted for sex, age			Adjusted for sex, age, education (years), morbidity
Employed	3645	60.88	1.00 (–; –)	3604	60.90	1.00 (–; –)
self-employed	205	61.95	1.02 (0.92; 1.14)	202	61.88	1.02 (0.92; 1.14)
Unemployed/benefits	213	46.01	0.76 (0.66; 0.88)	208	46.15	0.80 (0.69; 0.93)
Social welfare recipients	471	41.19	0.67 (0.60; 0.75)	453	41.50	0.77 (0.69; 0.87)
Others	93	43.01	0.71 (0.56; 0.89)	73	45.21	0.77 (0.60; 1.00)
Education (years)			Adjusted for sex, age			Adjusted for sex, age
< = 10	886	45.94	1.00 (–; –)	886	45.94	1.00 (–; –)
10–15	3415	60.59	1.32 (1.22; 1.42)	3415	60.59	1.32 (1.22; 1.42)
> 15	239	67.36	1.49 (1.33; 1.67)	239	67.36	1.49 (1.33; 1.67)
Income			Adjusted for sex, age			Adjusted for sex, age, education (years), occupational status, living alone
Low tertile (< 195.000DKK/year)	1526	47.18	1.00 (–; –)	1467	47.17	1.00 (–; –)
Middle tertile (195.000–255.000DKK/year)	1552	59.54	1.26 (1.18; 1.35)	1535	59.54	1.16 (1.08; 1.24)
High tertile (> 255.000 DKK/year)	1545	66.99	1.40 (1.31; 1.49)	1536	67.12	1.25 (1.16; 1.34)
Number of visits to dentist the last 5 years			Adjusted for sex, age			Adjusted for sex, age, education (years), occupational status, income
0–1	608	37.83	1.00 (–; –)	570	37.19	1.00 (–; –)
2–4	300	37.33	0.99 (0.83; 1.18)	285	37.19	0.99 (0.82; 1.18)
≥ 5	3697	63.00	1.64 (1.48; 1.82)	3665	63.08	1.53 (1.37; 1.71)
Morbidity			Adjusted for sex, age			Adjusted for sex, age, education (years), occupational status, income
No	3478	60.21	1.00 (–; –)	3407	60.46	1.00 (–; –)
Yes	1150	50.87	0.82 (0.77; 0.87)	1131	51.02	0.88 (0.83; 0.94)

Model 1: Age- and sex adjusted. For age-groups, Model 1 included only adjustments for sex.

Model 2: Model two included adjustments as in Model 1 and additional adjustments for potential confounding factors. The model on age was adjusted for sex, educational attainment, occupational status and income. Ethnicity and cohabitation were adjusted for age, sex, educational attainment, occupational status and income. Educational attainment was adjusted for age and sex. The model on income was adjusted for age, sex, educational attainment, occupational status and cohabitation status. Occupational status was adjusted for age, sex, educational attainment and morbidity. Models including morbidity and use of preventive health services were adjusted for age, sex, educational attainment, occupational status and income.

**Table 3:** Relative risk and 95% confidence intervals (CI) of attendance to the Check Your Health Preventive Program, estimated by Poisson regression with robust error variances. Morbidity and medication purchase.^a^

	Model 1			Model 2		
	N	Attenders (%)	Relative risk (95% CI)	N	Attenders (%)	Relative risk (95% CI)
Diagnosis, (ICD-10 code)^a^						
Diabetes, (DE10 to DE14)						
No	4588	58.09	1.00 (–; –)	4500	58.29	1.00 (–; –)
Yes	40	35.00	0.59 (0.39; 0.90)	38	36.84	0.64 (0.42; 0.97)
Hypercholesterolemia, (DE78)						
No	4612	57.94	1.00 (–; –)	4522	58.16	1.00 (–; –)
Yes	16	43.75	0.74 (0.43; 1.29)	16	43.75	0.75 (0.44; 1.28)
Asthma, (DJ45 and DJ46)						
No	4607	57.98	1.00 (–; –)	4518	58.21	1.00 (–; –)
Yes	21	38.10	0.64 (0.37; 1.11)	20	35.00	0.65 (0.35; 1.21)
Mental disease, (DF)						
No	4577	57.90	1.00 (–; –)	4488	58.13	1.00 (–; –)
Yes	51	56.86	0.98 (0.77; 1.25)	50	56.00	1.10 (0.86; 1.39)

In treatment with						
Antihypertensives last year						
No	4264	58.84	1.00 (–; –)	4176	59.12	1.00 (–; –)
Yes	364	46.70	0.75 (0.67; 0.84)	362	46.41	0.76 (0.68; 0.86)
Antidiabetics last year						
No	4550	58.22	1.00 (–; –)	4462	58.45	1.00 (–; –)
Yes	78	38.46	0.64 (0.49; 0.85)	76	38.16	0.67 (0.50; 0.89)
Antihyperlipidemics last year						
No	4448	58.21	1.00 (–; –)	4362	58.41	1.00 (–; –)
Yes	180	50.00	0.82 (0.70; 0.95)	176	50.57	0.87 (0.75; 1.01)
Asthmatic drugs last year						
No	4354	58.13	1.00 (–; –)	4267	58.38	1.00 (–; –)
Yes	274	54.01	0.92 (0.82; 1.03)	271	53.87	0.96 (0.86; 1.07)
Antipsychotics and lithium last year						
No	4524	58.42	1.00 (–; –)	4437	58.64	1.00 (–; –)
Yes	104	34.62	0.60 (0.46; 0.77)	101	34.65	0.76 (0.58; 0.99)
Anxiolytics last year						
No	4518	58.30	1.00 (–; –)	4428	58.54	1.00 (–; –)
Yes	110	40.91	0.68 (0.54; 0.84)	110	40.91	0.80 (0.64; 1.00)
Antidepressants last year						
No	4142	58.86	1.00 (–; –)	4062	59.08	1.00 (–; –)
Yes	486	49.59	0.82 (0.75; 0.90)	476	49.79	0.93 (0.84; 1.02)

Model 1: adjusted for gender, age.

Model 2: adjusted for gender, age, education (years), occupational status, income.

a ICD-codes represent the highest ICD-10 level with all sublevels included. Hypertension (ICD-10: DI11 to DI15) is not reported since no one had records of hypertension.
